# Penetrating Trauma-Induced Perilymphatic Fistula: A Case Report and Literature Review

**DOI:** 10.7759/cureus.36106

**Published:** 2023-03-13

**Authors:** Takahiro Nakajima, Masaomi Motegi, Yutaka Yamamoto

**Affiliations:** 1 Otolaryngology - Head and Neck Surgery, The Jikei University, Tokyo, JPN

**Keywords:** literature review, case report, hearing outcome, fistula repair procedure, exploratory tympanotomy, computed tomography, earpick, perilymphatic fistula, penetrating ear trauma

## Abstract

This article highlights the importance of early identification and surgical treatment for extremely rare traumatic perilymphatic fistula (TPF) caused by an earpick, which can pose the risk of irreversible hearing loss. Herein, we have described two cases of TPF and reviewed the literature primarily based on surgical treatment for penetrating ear trauma-induced TPF.

We highlight the case of two females who sustained an accidental penetrating injury in the ear caused by the introduction of an earpick, leading to hearing loss and dizziness. Pure tone audiometry detected elevation of the bone-conduction thresholds. Computed tomography of Labyrinth revealed pneumolabyrinth in one case. Both patients underwent exploratory surgery, we completely repositioned the stapes that had invaginated into the vestibule in one case, in the other case, we reconnected the disarticulated incudostapedial joint and sealed perilymph fistula caused by rupture of the oval window. Both patients achieved hearing improvement and complete relief from the vestibular symptoms.

The literature review indicated that a scar on the posterior aspect of the tympanic membrane was found in 44.4% of cases. Hearing improvement was observed in 45.5% and 25.0% of cases with invagination of stapes and fractured footplates by fistula repair, respectively. In terms of handling stapes dislocation, the hearing improvement rate was better in cases of complete stapes repositioning (66.7%) than those of complete or partial stapes removal (16.7%). Preoperative mild bone-conduction hearing loss or localized pneumolabyrinth are favorable factors for satisfactory hearing. When surgery is performed within 11 days of the injury, satisfactory hearing improvement can be expected.

## Introduction

Penetrating trauma through the ear can injure the middle ear structure, potentially leading to perilymphatic fistula. However, it can occasionally be missed or misdiagnosed because of the complicated signs associated with mixed impairment in the ossicles or inner ear. For cochlear dysfunction, once traumatic perilymphatic fistula (TPF) develops, sudden or progressive sensorineural hearing loss can manifest in the days after trauma [1,2].

Earpicks are sticks used for cleaning ear wax and are more popular in Asian countries. They are usually made of harder materials, including wood, metal, or plastic, and have a slightly sharper tip compared to a cotton swab. While inserting the earpick, unfortunately, forces the sharp tip into the ear canal, such as falling or someone hitting the arm, the tip can easily wound the deeper external ear meatus. In such cases, the middle ear structures are likely to be injured through the perforation of the tympanic membrane (TM). This trauma can simultaneously interrupt the ossicular chain, which poses the risk of stapes dislocation and resultant TPF; however, such cases are extremely rare, and reviews related to this topic are scarce.

We encountered two cases of TPF that required urgent exploratory tympanotomy. Further, the present article discusses the surgical strategy for penetrating trauma-induced TPF.

## Case presentation

Case 1

**Figure 1:**
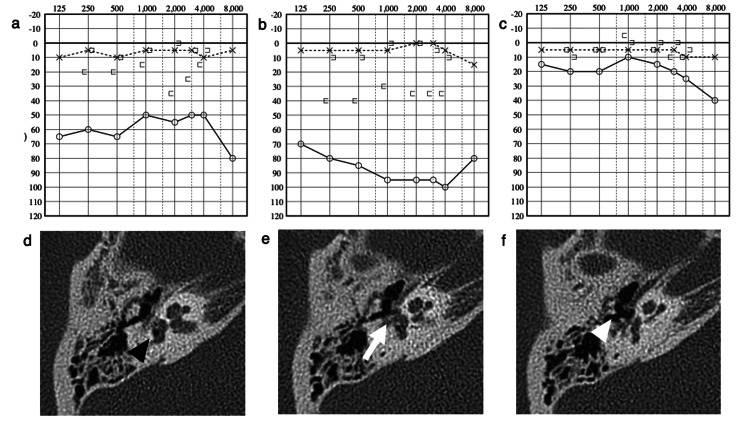
Perioperative audiograms and preoperative computed tomography (CT) images of Case 1 (a) Audiogram obtained at the time of admission shows a right mixed hearing loss. (b) Audiogram evaluated four days after admission illustrates worsening of both air- and conductive-hearing thresholds. (c) Audiogram three months after surgery demonstrates that hearing thresholds are almost recovered. (d) Axial planes of the preoperative CT image. Notably, an air density area (arrowhead) is shown inside the vestibule (pneumolabyrinth). (e) The incudomalleolar joint is not disarticulated, whereas invagination of the stapes into the vestibule is noted. Dislocated stapes (arrow) are noted. (f) The incudostapedial joint is disarticulated (arrowhead).

A 31-year-old woman sustained a forceful penetrating injury in the external ear meatus caused by an earpick while cleaning her ear. She visited a hospital the same day because of dizziness and tinnitus. Otoscopy revealed swelling of the posterosuperior aspect of the right TM without perforation. She had horizontal-rotatory positional nystagmus to the right. Pure-tone audiometry (PTA) detected moderate bone-conduction hearing loss (Figure 1a). The air density area in the vestibule (Figure 1d) and invagination of stapes into the vestibule (Figure 1e) were detected on high-resolution computed tomography (HRCT). These images also showed a disconnection in the incudostapedial joint (Figure 1f). Despite systemic steroids and antibiotics, her dizziness worsened, and the bone-conduction threshold deteriorated on day 4 after the trauma on repeated PTA (Figure 1b). An exploratory tympanotomy was performed five days after the injury. The incudostapedial joint was disarticulated and the stapes were invaginated to the vestibule. Perilymph leaked through the injured membrane in the oval window. No sign of leaked perilymph from the round window was observed. We gently pulled up the stapes using a hook until it was repositioned to the oval window and reconnected to the incudostapedial joint, not revealing the stapes fracture. The fistula was then sealed with connective tissue. The dizziness disappeared completely three days postoperatively. Her hearing remarkably improved three months after the surgery (Figure 1c). She was followed up for approximately one year after surgery, and no complication recurred.

Case 2

**Figure 2:**
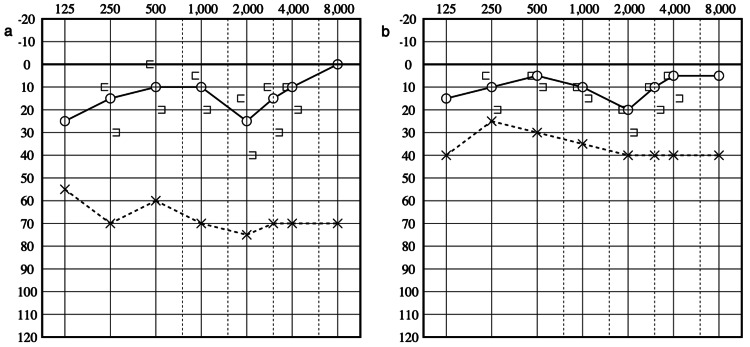
Perioperative audiograms of Case 2 (a) Audiogram obtained at the time of admission shows severe threshold elevation in air conduction with mild elevation of bone conduction threshold. (b) Audiogram evaluated 6 months after the surgery. The air-bone gap had decreased.

**Figure 3:**
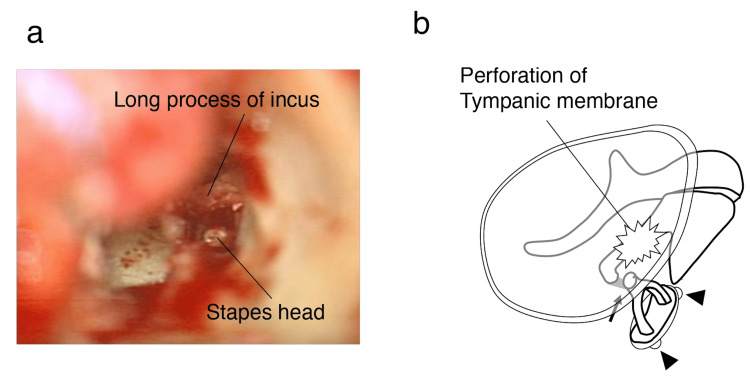
Intraoperative view and clinical image of the tympanic cavity Intraoperative view (a) and clinical image (b) of the tympanic cavity show the incudostapedial joint disarticulation and fold connection (arrow). The configuration of the stapes is completely preserved. Perilymph leaked from the oval window fistulae (arrowhead). Perforation in the posterosuperior aspect of the tympanic membrane is illustrated in the clinical image. Image credit: Takahiro Nakahiro

A 39-year-old woman accidentally thrust an earpick deep into the left ear canal while scratching there. She experienced dizziness immediately after the accident, prompting her to visit the hospital on the same day. Otoscopy revealed a perforation in the posterosuperior aspect of the TM. She had horizontal-rotatory gaze nystagmus to the left. The PTA revealed left mixed hearing loss (Figure 2a). Despite the fact that imaging assessment was not conducted, exploratory tympanotomy was performed because TPF was strongly suspected based on symptoms and sensorineural hearing loss. During the operation, the disarticulated incudostapedial joint connecting the folds was observed, whereas the general orientation of the stapes was preserved (Figures 3a, 3b). Perilymph leaked from the ruptured annular ligament of the oval window but not from the round window membrane. The perilymphatic fistula was closed by fascia, and the perforation of the TM was repaired using the underlay technique. Finally, the trimmed incus was placed between the head of the stapes and the TM. Dizziness disappeared seven days postoperatively. The TM perforation also closed completely. The patient was discharged, and her hearing improved six months postoperatively (Figure 2b). The patient was followed up for approximately one year after surgery, and her symptoms did not recur.

## Discussion

We present the cases of two patients with rare TPF caused by earpick injury who achieved a satisfactory outcome in hearing and vestibular symptoms following surgical restoration. Literature related to penetrating injury-induced TPF confirmed by surgery was reviewed (Table 1) [1-16].

**Table 1:** Characteristics of patients with perilymphatic fistula caused by penetrating ear trauma, including clinically suspected cases Abbreviations: AC, air conduction; ASQ, antero-inferior quadrant; BC, bone conduction; CRS, complete repositioning of the stapes; dB, decibels; FP, footplate; SS, superstructure; OP, ossiculoplasty; OPE, operation; PRS, partial repositioning of the stapes; PSQ, posterior superior quadrant; PTA, pure tone audiogram; RS, removal of the stapes; SO, sealing of the oval window; TM, tympanic membrane

Reference	Age (years) /sex	Cause	Injured location of TM	Interval to surgery	Pneumo- labyrinth (Location)	Stapes status	Surgical procedure	Threshold in pre OPE PTA (dB)	Improvement/ threshold in post OPE PTA (dB)	Postoperative vestibular symptom
Yamasoba (2003) [1].	13/F	Earpick	Posterior half	Unknown	Presence (Vestibule)	Deep invagination	RS, SO, OP	Conductive AC: 30 BC: unknown	Not improved AC: 45 BC: unknown	Complete relief
Herman (1996) [2].	47/F	Cotton tip	Unknown	3 years	Presence (Vestibule)	Deep invagination	Labyrin- thectomy	Mixed AC: unknown BC: unknown	Not improved	Complete relief
Vanderstock (1983) [3].	47/F	Knitting needle	PSQ	16 days	Unknown	Deep invagination	RS, SO, OP	Conductive AC: 55 BC: 18	Not improved AC: 45 BC: 45	Complete relief
Yanagihara (1987) [4].	48/F	Knitting needle	Unknown	4 months	Unknown	Stapes tilted anteriorly	CRS, OP, SO	Mixed AC: 41 BC: 44	Improved AC: 35 BC: 31	Complete relief
Snelling (2006) [5].	44/M	Metal hair band	Posterior	3 months	Presence (Vestibule)	FP fracture, fragment in vestibule	SO	Mixed AC: 84 BC: 55	Not improved AC: scale out BC: scale out	Unknown
Ishida (2006) [6].	35/M	Tip of comb	Intact	8 days	Presence (Vestibule)	Deep invagination fracture of FP	RS, SO	Mixed AC: 88 BC: 48	Improved AC: 29 BC: 15	Complete relief
Nishiike (2008) [7].	72/F	Comb	Posterior half	4 days	Presence (Vestibule)	Slight invagination	OP, SO	Mixed AC: 77 BC: 27	Not improved AC: 77 BC: 38	Complete relief
Hatano (2009) [8].	40/M	Earpick	Unknown	11 days	Presence (Vestibule)	Deep invagination	Stapes left in vestibule, OP, SO	Mixed AC: 107 BC: 57	Improved AC: 50 BC: 15	Partial relief
Ederies (2009) [9].	41/M	Temple pick of eyeglass	PSQ	2 months	Presence (Vestibule)	Fracture of FP and SS	Partial stapedotomy, SO	Mixed AC: 88 BC: 39	Not improved	Complete relief
Tsubota (2009) [10].	20/M	Earpick	PSQ	6 days	Presence (Vestibule)	Shallow invagination	CRS, OP, SO	Mixed AC: 104 BC: 67	Not improved AC: 90 BC: unknown	Complete relief
Hidaka (2012) [11].	85/M	Chop- stick	PSQ	32 days	Presence (Vestibule)	Inverted completely	RS, SO	Mixed AC: 102 BC: 67	Not improved	Complete relief
Bogaerts (2014) [12].	32/F	Head massage device	Anterior	9 days	Presence (Vestibule)	Shallow invagination	CRS, SO	Conductive AC: 38 BC: 18	Improved AC: 27 BC: 17	Complete relief
Ginat (2015) [13].	66/F	Stick	Intact	Unknown	Presence (Vestibule)	Deep invagination	RS, SO	Mixed AC: 80 BC: unknown	Not improved	Complete relief
Comacchio (2019) [14].	36/F	Earpick	ASQ	3 years	Presence (Vestibule)	FP fracture	SO	Mixed AC: 91 BC: 64	Not improved	Partial relief
Ren (2020) [15].	60/F	Earpick	Intact	4 months	Presence (Vestibule)	Dislocation	RS, SO, CRS	Mixed AC: 84 BC: 50	Improved AC: 67 BC: 57	Complete relief
Paramasivam (2020)[16].	50/M	Stick	Intact	3 months	Presence (Labyrinth)	Dislocation	CRS, SO	Mixed AC: 90 BC: 48	Not improved	Complete relief
Case 1	39/F	Earpick	PSQ	2 days	Unknown	Intact	SO	Mixed AC: 69 BC: 25	Improved AC: 39 BC: 20	Complete relief
Case 2	31/F	Earpick	PSQ	5 days	Presence (Vestibule)	Shallow invagination	CRS, SO	Conductive AC: 55 BC: 20	Improved AC: 18 BC: 4	Complete relief

Symptoms and clinical findings

All patients with penetrating trauma-induced TPF suffered from vestibular and cochlear symptoms, including conductive, sensorineural, or mixed hearing loss. In terms of otoscopic views, scars on the posterior aspect of the TM were present in 44.4% cases (Table 1). Accurate assessment for stapes dislocation is often challenging even when evaluating by HRCT, often due to partial volume effects. Therefore, TPF should be suspected when injury surrounding the posterior TM occurs, particularly when persistent trauma-induced vestibular or cochlear disturbances are noted, regardless of the presence of any findings on CT.

Role of computed tomography

HRCT can confirm the configurational abnormality of stapes, such as the stapediovestibular luxation or fractures. In typical external ear trauma, stapes dislocation is rarely encountered because the stapes is protected from direct injury by the overhanging scutum [17]; however, this dislocation is frequently observed, particularly in 94.4% of penetrating injury-related TPF (Table 1). This high prevalence is partly plausible because earpicks can cause deeper penetration into the middle ear cleft. Therefore, CT should be carefully evaluated with special attention to refine the stapes dislocations. Furthermore, HRCT is also highly sensitive to pneumolabyrinth, which is indirect evidence of perilymphatic fistula, observed in 83.3% of prior descriptions (Table 1). Although HRCT assessment is useful for early TPF diagnosis, the findings of Case 2 indicate that CT is not a prerequisite for this diagnosis; therefore, other factors, such as hearing results and vestibular symptoms, should also be comprehensively deliberated for this diagnosis.

Indication and optimal time to surgery

Currently, there is no specified protocol towards surgical intervention for TPF. Given the lack of definitive diagnostic criteria in practice, conservative treatment is initially preferred. Although there may be an impact of publishing bias, most published cases ultimately progressed to surgical treatment. Contrary to idiopathic fistulae, disrupted annular ligaments or fractured footplates in TPF may cause more copious perilymph leakage, eventually leading to complete inner ear dysfunction [3]. Therefore, TPF is likely to require early surgical correction.　Fundamentally, we follow this strategy: surgery should be performed immediately once traumatic perilymphatic fistula is suspected. Meanwhile, controversy exists regarding the optimal period for surgery. In Case 1, worsening symptoms and imaging evidence of invagination of the stapes prompted us to perform surgical exploration within five days of trauma. Our review indicated that bone-conduction hearing outcomes were satisfactory when surgery was performed within 11 days of injury [6,8,12]. Another study similarly demonstrated that hearing was restored after surgery when performed within approximately 10 days of injury, unless irreversible severe hearing loss had occurred [10]. From these studies, it can be concluded that even when physicians misdiagnose or have compelling reasons to select a conservative treatment approach for several days during the course of therapy, satisfactory hearing prognosis may still be expected with surgery.

Repair for injured stapes

The repair method for dislocated stapes in TPF remains unstandardized and variable, without achieving consistent hearing outcomes. Table 1 shows numerous surgical procedures, including stapes repositioning, leaving the stapes in the vestibule, stapedectomy, and labyrinthectomy. The type of stapes injury did not predominantly impact hearing prognosis. Regarding features of stapes injury, hearing improved in only 45.5% and 25.0% of cases with stapes invagination and its fractured footplates, respectively. In terms of procedures for stapes management, hearing improved in 16.7% of cases with complete or partial stapes removal compared to 66.7% with complete stapes reposition. Even in with a deeply invaginated stapes, complete stapes repositions resulted in satisfactory hearing [15].

There has been controversy in treating invaginated stapes. One surgeon advocated that removal of the invaginated stapes should be avoided due to risk of further sensorineural hearing loss [3]. Meanwhile, others promoted the repositioning of depressed stapes [18,19] because leaving the stapes in the vestibule is presumed to cause late irreversible cochleovestibular dysfunction owing to the scarring in the vestibule or persistence of perilymphatic leakage [2]. We also pulled the depressed stapes to its original position uneventfully, possibly preventing further inner ear dysfunction. As evidenced by the better hearing outcomes in cases with complete stapes repositioning compared to those with stapes removal in our review, it is assumed that meticulous repositioning of the stapes does not adversely affect hearing outcome even in TPF with a deeply invaginated stapes.

Comorbid sensorineural hearing loss

The hearing outcome may be related to the severity of preoperative sensorineural hearing deterioration. Our cases and the review support the notion that satisfactory hearing outcome is anticipated for TPF with a mildly elevated bone-conduction threshold [10]. Meanwhile, even when preoperative sensorineural hearing deteriorated moderately or severely (preoperative bone-conduction threshold ≦ 50 dB), bone-conduction restoration was achieved in 33.3% cases (Table 1). In practice, there has been no methodology to determine whether such sensorineural hearing loss is irreversible or not; thus, in the early period after trauma, surgical hearing restoration is advisable even for TPF with moderate or severe sensorineural hearing loss.

Pneumolabyrinth

A critical sign of perilymphatic fistula is pneumolabyrinth, which was observed in 83.3% of cases (Table 1). Aeration inside the scala vestibuli can induce destruction of the membranous labyrinth, leading to irreversible sensorineural hearing loss. The prognosis related to pneumolabyrinth differs depending on its location [20]. When air invasion occurred solely inside the vestibule or semicircular canals, more than half of those with hearing impairments recovered, whereas when it occurred in both the vestibule and cochlea, improvement was infrequent. Nevertheless, as shown in Case 1, the presence of pneumolabyrinth alone does not impact hearing prognosis (Table 1).

Meanwhile, the presence of pneumolabyrinth is not indicative of active TPF. Some researchers [11,12] have described that conservative treatment resulted in hearing improvement in TPF with radiologically confirmed stapedial dislocation and/or pneumolabyrinth. A stable hearing level and improvement in vestibular symptoms can justify conservative treatment with close monitoring of hearing even in patients with TPF with pneumolabyrinth.

Limitations

 First, publication bias was evident in our review. When a favorable prognosis was not expected, clinicians generally did not recommend surgery. This might limit the generalizability of our review to patients who do not undergo surgery because of profound hearing loss. Second, exploratory tympanotomy is mandatory for diagnosis of TPF; however, surgical indications may be specific to each center. Therefore, cases managed with conservative treatment may have been unintentionally excluded from our review, resulting in the exclusion of undiagnosed TPF.

## Conclusions

Persistent or progressive vestibulocochlear symptoms after a penetrating trauma should raise suspicion of TPF. Worsening symptoms, a scar on the posterior aspect of the TM, or stapes dislocation or pneumolabyrinth on CT can help in the early identification of TPF. Preoperative mild bone-conduction hearing loss or localized pneumolabyrinth can be favorable factors for satisfactory hearing outcomes. Although the impact of stapes injury type or its repair method on hearing prognosis remains uncertain, complete stapes repositioning may be considered, even for an invaginated stapes. Surgical repair of the fistula should be considered immediately once TPF is suspected. Even for TPF with moderate or severe sensorineural hearing loss, surgical hearing restoration should be expected within approximately 10 days of injury.

## References

[1] Yamasoba T, Amagi N, Karino S (2003). Traumatic luxation of the stapes into the vestibule. Otolaryngol Head Neck Surg.

[2] Herman P, Guichard JP, Van den Abbeele T (1996). Traumatic luxation of the stapes evidenced by high-resolution CT. AJNR Am J Neuroradiol.

[3] Vanderstock L, Vermeersch H, De Vel E (1983). Traumatic luxation of the stapes. J Laryngol Otol.

[4] Yanagihara N, Nishioka I (1987). Pneumolabyrinth in perilymphatic fistula: report of three cases. Am J Otol.

[5] Snelling JD, Bennett A, Wilson P, Wickstead M (2006). Unusual middle-ear mischief: trans-tympanic trauma from a hair grip resulting in ossicular, facial nerve and oval window disruption. J Laryngol Otol.

[6] Ishida K, Sakai M, Hiroi M, Sekine M, Takeo T (2006). Traumatic fracture of the stapes and perilymph fistula: report of a case. Tokai J Exp Clin Med.

[7] Nishiike S, Hyo Y, Fukushima H (2008). Stapediovestibular dislocation with pneumolabyrinth. J Laryngol Otol.

[8] Hatano A, Rikitake M, Komori M, Irie T, Moriyama H (2009). Traumatic perilymphatic fistula with the luxation of the stapes into the vestibule. Auris Nasus Larynx.

[9] Ederies A, Yuen HW, Chen JM, Aviv RI, Symons SP (2009). Traumatic stapes fracture with rotation and subluxation into the vestibule and pneumolabyrinth. Laryngoscope.

[10] Tsubota M, Shojaku H, Watanabe Y (2009). Prognosis of inner ear function in pneumolabyrinth: case report and literature review. Am J Otolaryngol.

[11] Hidaka H, Miyazaki M, Kawase T, Kobayashi T (2012). Traumatic pneumolabyrinth: air location and hearing outcome. Otol Neurotol.

[12] Bogaerts M, Waterval J, van Dinther J, Somers T, Zarowski A, Offeciers FE (2014). Treatment of traumatic stapediovestibular luxation: case report with the introduction of a new technique and review of literature. Otol Neurotol.

[13] Ginat DT, de Venecia RK, Curtin HD (2015). Stapediovestibular dislocation depicted on temporal bone computed tomography with 3D rendering. Am J Otolaryngol.

[14] Comacchio F, Guidetti G, Guidetti R, Mion M (2019). Pneumolabyrinth and recurrent paroxysmal positional vertigo after traumatic stapes fracture. Ann Otol Rhinol Laryngol.

[15] Ren Q, Su J, Si J, Li Y, Ding X (2022). Traumatic stapediovestibular dislocation with intact stapes: report and review. Ear Nose Throat J.

[16] Paramasivam S, Ramasamy K, Alexander A, Saxena SK (2020). A rare cause of vertigo - case report and review of literature. Glob J Otolaryngol.

[17] Meriot P, Veillon F, Garcia JF, Nonent M, Jezequel J, Bourjat P, Bellet M (1997). CT appearances of ossicular injuries. Radiographics.

[18] Arragg FG, Paparella MM (1964). Traumatic fracture of the stapes. Laryngoscope.

[19] Hakuba N, Iwanaga M, Tanaka S (2010). Ear-pick injury as a traumatic ossicular damage in Japan. Eur Arch Otorhinolaryngol.

[20] Kobayashi T, Sakurada T, Ohyama K, Takasaka M (1993). Inner ear injury caused by air intrusion to the scala vestibuli of the cochlea. Acta Otolaryngol.

